# Editorial and Miscellaneous

**Published:** 1863-07

**Authors:** 


### EDITORIAL AND MISCELLANEOUS.

### CALOMEL IN THE ARMY.

THE SURGEON-GENERAL'S ORDER.

The recent action of Surgeon-General Hammond, cutting off Calomel and Tartar Emetic from the Army Medical Supply Table, has given rise to a great deal of both professional and unprofessional comment, and an abundance of both pertinent and impertinent remark. The secular press have grown oracular upon it; the medical press, in greater part, have touched the matter gingerly, if at all; whilst one or two have expectorated upon the order their direst wrath. A number of medical gentlemen in this city, numbering about the detailed squad at a major's funeral, in the name of the American Medical Association, (*Quantum mutatus ab illo!*) censured it in language as mild as a skim-milk projectile; whilst an indignant society in one of our sister cities, exhausts the vocabulary of Billingsgate in characterizing it, and only pauses in piling up still darker adjectives from positive exhaustion. All the notes of opposition have been sounded, from the deep-toned base of blackguardism up, through all the gamut of scurrility, to the highest tones of vituperation and invective.

The senseless cachinnation of quackdom, from the rough adherent of Sam. Thomson glorying in his fool's cap and bells, to the dainty and emasculated votary of Hahnemann with his dreary spiritualistic platitudes and shadows, may be passed over as wholly outside of the argument. What they cackle, or ethereally suspire, weighs not a feather in deciding this question. Great principles depend neither on the wise man's nod, nor the fool's grin. A great action is none the less to be admired because the mob throw up their hats and applaud—it is none the less to be investigated because they contemn it. As Medicine is above popular prejudice, it is also superior to its prepossessions. The questions it discusses are original, independent questions.

We confess that when we first heard of this order, which, by the way, was from the lips of our correspondent of last month, we were not only surprised but disposed to believe it an error in judgment on the part of the Surgeon-General. We are not yet certain that the object sought might not have been secured in a manner less displeasing to the profession at large. It seemed to detract something from that proud professional independence which had always been our boast. Whatever our opinion of the merits or demerits of any particular agent, or whatever the opinion of our patients in the premises, we have always claimed and shall continue to claim the strictly scientific and professional right to use it, or let it alone, in any particular case, or series of cases, which may arise in our practice. What we want in practice is potential agencies—we do not believe in specifics or panaceas, and he who undertakes to crowd them down our throat is as repulsive to us as he who takes away the power which we wish to use. But we waive that point at present.

The readers of this Journal know what our views are with reference to Mercurials, and both they and the students of Rush Medical College know that we recommend their use, under such limitations as have been pointed out by the more exact observations of modern time. Of Tartar Emetic we only say, now, that after observation of its use compared with that of Veratrum Viride, for many years, we give the Antimonial largely the preference—though, truth to say, we use neither as often as in the earlier days of our practice. But if the use of Mercurials and Tartar Emetic is to be the criterion of orthodoxy—we certainly are orthodox—at least occasionally. With advancing years we confess, with deep contrition, we are getting to depend more and more upon the due regulation of the "*non naturals*," air and water, food, exercise and sl ep, than we do of the cunningest combination of drugs. We are getting to think vastly less of *spanæmics* and vastly more of nutrients—much less of *alteratives*, and much more of those things which energise nutrition and directly sustain the failing powers of life.

When under the excitement of the discovery by sundry North-eastern gentlemen of the remarkable powers of Quinine over certain forms of endemic disease, long well known in the West and South, they undertook to make it a panacea and omnipotent prophylactic, and by official order to crowd millions of ounces down the throats of the unhappy soldiery, this Journal urged its indignant protest and almost alone enunciated the true doctrine. Time, and a little experience have cooled the ardor of the apostles of Quinine-prophylaxis, and the excessive use of this most useful article has now shrunk to narrower limits.

The experiment was expensive—the resulting evils prompt in making themselves known. When we urged that better dietetic and general hygienic measures must be adopted to ward off pestilence from the army, they ridiculed the idea, and urged the purchase of more Quinine.

There were "many dry eyes" at the interment of "Quinine-prophylaxis." There are a multitude more dry eyes as Surgeon-General Hammond musters the mourners at his recent order.

We said we thought at first that the Surgeon-General might have adopted some course less distasteful to the profession. Reflection convinces us that from his point of view he could scarcely have taken any other course than that which he has taken. And this conclusion has been arrived at only after mature consideration.

In the first place the indignation expended on the Surgeon-

General is from a mistaken idea of his position, and the position of his subordinates on the medical staff.

The Surgeon-General is responsible for the success of his department, for the health and lives of the soldiery, as a commanding general is for the success of the army in the field. He is placed in the position of one who has to accomplish through others that for which he is held to personal accountability, as the private practitioner is for his own methods of treatment. It may be that there are private soldiers better informed and with better judgment than the officers over them—officers also superior in qualifications to their generals and commander-in-chief—but the usages of war and the necessities of discipline require that the private judgment of the inferior, in arbitrary rank, shall be wholly subservient to the superior. And this is not deemed improper or entailing any disgrace to the subordinate. On the contrary, implicit obedience to orders is the highest virtue of the service. The army is a vast machine responsive to a single master hand, and the master is solely responsible.

But this is too clear to need argument. The Surgeon-General is General over his subordinates, and is held responsible for them, and hence has clearly a right to control their official actions according to the dictates of his own judgment, not according to their's, or your's, or mine.

Is a great profession about to denounce one of their number, upon whom such tremendous responsibilities rest, for using or not using this or that agent, whilst each of its members claims the absolute right to use or not to use them as he pleases?

In joining the army, observe, the surgeon has merged his personality in it, and become only a part of the great engine of power. He is only responsible for obedience to the order which comes down to him, whereas the Surgeon-General is responsible as an individual, and has the rights of an individual to employ or not to employ, by himself or his agents, whatever he in his best judgment thinks best.

But it is said he has based his action upon false premises, and therefore is censurable. His primary expressed reason for the removal of Calomel and Tartar Emetic from the Supply Table is, that they have been abused in use and that the consequences have been disastrous—a statement which the Chicago meeting insinuated to be unproved, and the Cincinnati brotherhood roundly asserted to be libelous. Here is a simple question of fact, and by the record only is it to be proved or disproved. Where is the record? In the hands of the Surgeon-General. On the one side stands the official record—vouched for by a professional gentleman of high distinction, thoroughly versed in the scientific methods of observation and analysis of the present age; a man against whose personal integrity his bitterest official enemy dare not breathe a whisper, and who has responsibilities piled upon him beyond any which the world has ever known, (for we repeat what we have elsewhere said, that his position is infinitely more important than that of any mere general in the field), and on the other side we have but the irresponsible *say-so* of individuals of perhaps private civil eminence, and the prejudices and prepossessions of persons of limited view who fancy that the removal of a couple of agents, from the medical *armamentaria*, is about to break down all divisions between scientific and pseudo-medicine and bring the quack on the level with educated physicians.

If any one knows the truth of this premiss, we insist, it is the Surgeon-General, and we are bound as honorable members of the profession to accept his statement to be true until, by evidence fully as authoritative as that which he claims to have in his bureau, the opposite is made to appear.

We apprehend that no educated physician will assume that his second statement, viz: that the advance of modern pathology has narrowed the range of useful application of the two mooted agents, is at all questionable.

For example, the old idea of Calomel was that it is an "antiphlogistic," hence applicable in all febrile and inflammatory affections—all educated medical men now know that it is not an antiphlogistic, but rather a *stimulant*, rapidly disintegrating the tissue, exhausting by excessive cell and molecular change.

True, by its primary action as a cathartic it may prove antiphlogistic, but every repetition of the dose develops more and more the stimulating i. e. tissue-disintegrating effect.

Why not then, it may be asked, use it as a stimulant? Because it rapidly exhausts without replacing tissue. That stimulant is always to be chosen which is adjuvant to increased nutrition; a part, or the whole, of which is capable of entering into and forming a portion of the organism.

But we do not propose to discuss the action and uses of Calomel. Practicing upon our own responsibility we use it when we choose, and shall continue to do so, because no one else is responsible. If we had voluntarily placed ourselves where some one else was responsible, we should either conform to his directions or resign. There is not a surgeon in the army who will permit his assistants, or the Hospital Steward, to use what he has directed not to be used.

Now if the Surgeon-General honestly entertains the views he has laid down in his circular, with regard to the abuses prevailing in the employment of the articles named, and the present restricted range of its application all admit, we submit that he has a clear and indefeasible right, nay it was his duty, to issue such an order, and the attempt to ostracize him from the profession in any quarter will recoil upon the projectors. It is an abandonment of the grand principles of toleration, which are the true glory of our profession.

Is there "a divinity which doth hedge around" these two remedies, that renders it indispensable for them to be placed upon the Supply Table? We have heard it said boastingly that at least two grand divisions of the army have enough of each on hand to last for two years—a fact probably as well known to the Surgeon-General as to those who boast of it. If, as is claimed, he proposed by the order to entirely "suppress" the use of them, why did he not couple with the notice of striking from the supply table, an order that the amount on hand should be returned at once to the Medical Purveyors of the different districts? Or why did he leave the matter remotely inferential, and not directly order that no more should be used? If he was actuated, as some claim, by an affiliation with the "Eclectics," and a leaning towards Homœopathy, why did he not strike off all the Mercurials, Arsenic, Iron, Iodide of Potassium, &c., at one fell swoop? Blue Mass, Corrosive Sublimate, the Iodides of Mercury, &c., all still remain. Does not this prove, if further proof were wanting, (which is not,) that this is not a mere senseless concession to "Anti-Mineral" knaves and fools, but a conscientious effort to remedy a great evil—wide spread, yet entrenched in prejudices and wrapped about with a cloud of "authorities?" Practically who does not know that it is by far too common a practice to give Calomel as a *placebo*—to give it when in doubt as to correct treatment—for whatever the disease may turn out to be, in digging among the fossil strata of the libraries, one can always find "authority" for its use. As a genial professional brother at our elbow suggests, some give it as the gamesters say is their rule—*when in doubt they play trumps.* Humiliating as this may be to the pride of the educated, thinking physician, "it's true, 'tis pity, and pity 'tis, 'tis true."

Again, this is merely a local and temporary regulation. The Surgeon-General knows, as we all know, and even Cincinnati, insane upon the subject of Calomel as it is, admits it,—that the vast majority of diseases prevalent in the army utterly forbid the use of Calomel and Tartar Emetic. They are either Scorbutic, Typhoid or "Malarious" in their type—one or more, requiring restorative, tonic, stimulant and nutritive methods, and not these potent agents. The exceptions to this rule are few and far between.

A vast number of the army surgeons are young men not as yet grounded in practice, guided mainly by their text books; another large class are men advanced in years, wedded to their particular notions, many of which are dogmas of the most baseless sort. Besides these we admit the presence in the army of very many both young and old, fully up to the times—sound physiologists, thorough students of hygiene, and *therefore* good practitioners. The latter class did not need this order—for the first two classes it was indispensable. But an army order must be general in its terms—it must be public in its issue.

It has been said that the first two classes, if not competent to administer these drugs with discretion, should have been discharged from the service, thus cutting the gordian knot of the difficulty. To those little familiar with the subject this would seem easy enough, but a mere trifle of thought will show its fallacy. The army surgeon has rights as well as duties. In the first place a court martial would be required in each case, else gross injustice might be done to individuals—and these courts-martial would necessarily, from the number and the important questions involved, be beset with infinite difficulties, besides taking away from their active duties medical officers who could not be spared from the service.

Or if they could be spared, how could charges be brought? Shall the production of a salivation be cause of dismissal, or collapse from the use of the Antimonial? How readily will the surgeon show that he used neither agent in inordinate doses, or with unwarrantable (text book and civil practice) continuance. And besides these surgeons are, perhaps, beyond reproach in every other respect—invaluable to the service. Are other military officers dismissed the service because they do not comply with orders never issued? In other branches of the service, we take it, when an evil exists the evil is corrected by a special order, and if the order is not obeyed then the offender is appropriately disciplined.

Can you order a surgeon not to salivate a soldier to whom he gives Calomel? The absurdity of the proposition is sufficient answer. But in this age of the world are we to be told that striking Calomel and Tartar Emetic from the Army

Supply Table leaves the surgeon so powerless that it is his duty rather to resign than to submit? We have yet to learn that the physician's duty, either in whole or even in greater part, consists in the administration of drugs. We believe with Cabanis, that there would still remain an art of medicine, invaluable to the world, were all, so called, medicines prohibited. It is the glory of our science that as it advances step by step, it becomes more powerful when applied to practice to palliate and remove disease by removing causes and changing conditions—placing the patient, briefly in normal physiological relations. It is the crowning triumph of the art, when the physician restores the patient to health, employing only those forces and those materials wherewith the Grand Architect builds up the human body.

Such extreme tenderness and sensibility, as is manifested in some quarters at the dropping of Calomel and Tartar emetic from the supply table seem to us to indicate on their part a want of confidence in medicine as a science, and goes far to bolster up the quacks of every hue in their anathemas of "regular" practice as mainly consisting in the exhibition of these two agents. As for us we have that degree of confidence in medical science as a whole, that if we were cut off entirely from the use of these articles we could still practice medicine with confidence and zeal. Why should these two bestride the medical world like the legs of a colossus? It is to be feared that too many yet carry them as Sinbad carried the Old Man of the Sea. We should be the masters and not the slaves of medicines, whatever their potency.

If the army was now engaged in a war under such conditions that alcoholic stimulants were found highly prejudicial, would it be unprofessional or "an insult to the profession" to order them dropped from the supply table?

As for ourselves, for instance, if we were the Surgeon General and we found that the surgeons were adopting the treatment recommended by Dr. Todd for fevers and phlogoses, we should be tempted to drop alcohol from the supply table.

And when we knew of the wretched results of the quinine madnia, before alluded to, we feared that an order on that subject might become necessary.

But Alcoholism and Quininism in their results are so open and palpable, that the merest modicum of good sense speedily corrects the practice of even the most bigoted dogmatist. The articles now stricken out are more dangerous, in the irregular life of the soldier, because the immediate results are less open and less palpable. As a matter of fact, it is perhaps safe to say, more salivations occur outside the camp hospitals than within them, for within the patient is protected from exposure and his own carelessness.

But suppose the Surgeon-General had, as some suggest would have been the better course, issued a circular cautioning his subordinates against the injudicious use of these agents, would there have been a particle less of sensation created, or less opposition begotten than is now present? Would not the quacks equally have gloried in it? It is our firm conviction that it would have been wholly unnoticed and disregarded by those to whom it was addressed. But now when called upon to face the fact that sooner or later they must face an empty Calomel bottle, and the extraordinary nature of the order itself, combine to bring the surgeon (as it should the civil practitioner) to reflect whether there may not be after all methods of cure superior to the Calomel and Tartar Emetic one to which he has been accustomed. He only needs to investigate, and he will find that the methods of treatment have advanced a wonderful distance from the Calomel and Tartar Emetic base of operations.

We know to day in this city excellent professional gentlemen who if they could be induced to leave out Calomel and its congeners from their practice for a few months, could scarcely be persuaded to take them up again, even in cases which positively demand their use.

Largely in this city, but beyond all in Cincinnati, are the deplorable results of the belief that Calomel is the central sun around which the whole word of medicine revolves, to be observed in most startling development.

The attempt at creating an excitement over this subject, will not advance the regular profession the dimension of a hair in popular repute, but will only confirm the vulgar opinion that Calomel is all there is of it. Vehement invectives will only convince that "it is the wounded bird that flutters."

How much better for the profession to plant itself, as it triumphantly can, upon the proud position that it is independent of special remedies, panaceas and specifics—that it can dispense with the use of a multitude such, and yet be itself indispensable and inestimable to the race.

Let the medical societies and associations confine their attention to the legitimate business of bringing out the facts, and developing the principles of medical science, and no more engage in the pitiful work of acting as star chambers consigning rebellious individuals to the rack, the guillotine and oblivion.

The attempt so far as Surgeon-General Hammond is concerned will fail—nay, it has already failed. Still young, energetic, far seeing, sagacious, independent and bold, he will carry the medical corps of the grand army which he has organized from chaos, and vivified from imbecility, not only through the trying ordeal of this unhappy war, but surround it with such a prestige and renown, that the whole profession will acknowledge the grand impulse which has been given them by one live, wise, earnest, discreet and PROGRESSIVE MAN.

---

*Spina Bifida.*—Mr. T. SMITH reported to the Pathological Society of London the removal of the Sac of Spina Bifida by operation. It was isolated from the spinal canal by a clamp. On the nineteenth day it was punctured. The clamp was screwed up close, the tumor was shaved off, and the edges touched with heated wire.

*Bobus.* We think it was Dr. Johnson who querulously retorted to one of his listeners, who was pestering him for explanations, that although he felt bound to furnish men *ideas*, he was *not* bound to furnish them *brains*. We were reminded of this the other day when a professional friend remarked to us that the following paragraphs in our last No. needed explanation:

"Our readers will bear us witness, that from the hour Nurse Yates sent a communication to the *Chicago Tribune*, calling upon the benevolent for a donation of rags to absorb the abominable flux from the salivation of the soldiery at Cairo, we have ever opposed this infernal practice in the army.

In our place in Rush Medical College, as teacher of the Practice of Medicine (and we know the Professor of Surgery has done the same), we have inculcated principles wholly at war with this prostitution and abuse of these energetic remedies."

Our friend tells us that some parties really seem to think that by these words levelled against "this infernal practice in the army" we deliberately denounce all *use* of Calomel in practice, where the infernal salivation is not produced! And again that "having inculcated principles wholly at war with *this prostitution and abuse* of these energetic agents" therefore our teachings in Rush Medical College are necessarily against their use in all cases whatsoever!

All we have to say to Bobus is, that he has revealed to us a depth of stupidity which we had not yet sounded. We commend you, Bobus, to your dictionary and some moderate study of the vernacular.

Machiavel says there are three degrees of capacity: "One man understands of himself, another understands what is explained; a third understands neither by himself, nor by any explanation; the first is excellent, the second commendable, the third altogether unprofitable."

We believe that Bobus belongs to the third class, and there we leave him. God help you, poor Bobus!

*Humors of the Craft.* A case of *Purpura haemorrhagica* is sent up to our table wherein the "M. D." advised and applied a string around the little finger and a string around the little toe of the sinister extremities to "suppress" epistaxis. No other treatment was suggested.—A Brigade Surgeon—"home from the wars"—and a proud evidence of the fact that homoeopathists can get into the grand army, (as the "secesh" do, in stolen uniforms,) to our own knowledge, a few days since was treating, what he asserted to be, a transverse fracture of the patella by fixing the leg in one of Day's double inclined planes.—Another who wished to rectify the deformity of a badly united leg, was lastly seen soaking the unhappy extremity in a tub of water "to take out the stiffening, so he could bend it into its place."—Cincinnati is in an uproar unequalled since the days of the Ephesians. The brethren verily believe their craft is in danger, because Surgeon-General Hammond refuses to pay for any more Calomel or Antimony for "our common Uncle's" soldiers. We suppose the order a "military necessity." No other ordnance can be used to kill the enemy, whatever its "potency" than that which "Uncle Sam" prescribes—why should he not also establish what ordnance only may be used to deplete his own army? Certain kinds of knives only are provided the surgeons—there certainly ought to be an indignation meeting, and the Surgeon General called upon to resign at once because he does not furnish some other patterns. But we forget "the Surgeon General is only a Physiologist"—true, a most eminent one, but even this is "very tolerable and not to be endured." What business has he to know anything about Physiology—the very essence of his business, and still stay in the army, where a man notoriously has the best places for knowing nothing of his business? Poor Hammond! He *writes* his name instead of having a mark to himself like an honest plaindealing man! Away with him, and hang him and his Physiology together!

*Duke.* One of these men is Genius to the other;
And so of these; which is the natural man,
And which the Spirit? who deciphers them?

*Dro. S.* I, sir, am Dromio; command him away,
*Dro. E.* I, sir, am Dromio; 'pray let me stay.—*Comedy of Errors.*

---

It is stated that what was called the "Medical Department of Lind University" is hereafter also to be called the "*Chicago Medical College.*" What is in the wind with the Reform School? It is not the first time that we have heard of the convenience of an *alias.*

---

*DeLaskie Miller, M. D.*, Prof. of Obstetrics and Diseases of Women and Children in Rush Medical College is at present visiting Europe with the object of adding to the interest of his department such means of illustration and practical improvements as may be commended to his judgement on personal observation of the scientific treasures of the old world.

We scarcely think it will be deemed against the dictates of the most delicate sense of propriety to say of Prof. Miller, in his absence, that although not beyond the middle period of life, he has already attained, not only the reputation but, the fact of thorough and extensive professional scholarship which has been corrected and invigorated by a range of practical experience in his department, at least, second to no other in this great city. Quick perceptions, a ready and retentive memory, ease and fluency in speaking, with the most conscientious anxiety to impress what he believes to be scientific truth upon the minds of students, render him deservedly a favorite of the medical classes, whilst his amiable, unassuming and gentlemanly demeanour, combined with remarkable practical tact, have commanded the most implicit confidence and personal regard of his numerous patients. Young, enthusiastic and devoted to his profession, we predict for him a career which will well repay his energy and assiduous industry. We hope to greet him on his return in September next, renewed in health and richly laden with professional good things which he will have " annexed " in his transatlantic tour.

---

*University of Michigan.* It is known to many of our readers that for a number of years past, there has been a constant effort on the part of the Homoeopathists to foist a chair of their absurdity upon the Medical Department of the University of Michigan. Although repeatedly defeated before the Board of Regents, they have been constantly stimulated in other attempts by the presence in the presidential chair of a zealous adherent to their doctrines. This person had infested the position from the time of his transfer from the charge of a seminary for young ladies in Bleecker St., New York City, some ten years since, until June the present year. At the time of his accession to the presidency, a position secured wholly by fraudulent practice upon a previous appointee, the income of the University was only some ten or eleven thousand dollars a year, but, in consequence of the rapid sale of the University lands, a few years later it was enhanced to some forty or fifty thousand. This vast amount so increased the facilities of the institution, that it could not escape growth in numbers—and this growth the President and his friends adroitly attributed to him, whilst it was really in spite of him. Nevertheless it kept him in the place an unexpected length of time. Arrogant, pompous, supercilious, overbearing and insolent, he long since disgusted all sensible people in Michigan, but managed to retain position from fear on all sides that the University could not stand another shock in its appointments. Such creatures always have a still lower grade of animals who toadey them. With just learning enough to amaze the groundlings, and just skill enough to plagiarize without discovery by the most casual readers, he has strutted and fumed a whole decade upon the Ann Arbor stage. Following out a precedent which he had himself initiated and established, the Board of Regents, with no prefatory self-exculpation, commended the chalice which he had long since poisoned, to his own lips, and he lies now upon the University boards, just back of the footlights, a mere carcase, beyond relief even from the infinitesimals of his cherished medical advisers.

We congratulate the Board of Regents upon this truly righteous step, demanded not only by the interests of the institution committed to their charge, but by common justice and the fitness of things.

And you, HENRY P. TAPPAN, D. D., "*Chancellor*," as you loved to call yourself, you thought that old "assassination did trammel up the consequence!" Apostle of little things—capable of the least of things—what is your present belief of Star Chamber justice?

One is dead—*de mortuis nil &c. &c.* H. P. T., D. D., &c., "packing up his books" returns crestfallen to Bleecker St.! And now only D——s remains!

> "The mills of the Gods grind slowly,
> But they grind exceeding small!"

---

"*Botanic*" *Druggists.* A correspondent calls our attention to the fact that we have among us divers apothecaries commited to the "eclectic" *alias* Thomsonian persuasion. Several notable instances have come to his knowledge, wherein the influence of these gentry has been employed to disparage the prescriptions of physicians who have unthinkingly sent patients to their shops. Our correspondent urges that it is as derogatory, to the interests and respectability of the profession, to patronize such establishments, as to consult with the quacks who are their especial pets. He directs the attention of the physician to the fact either that his prescription will not be honestly compounded, or that his patient will be practiced upon to the detriment of his confidence.

One concern in this city is especially alluded to, where the proprietor, already too much patronized by physicians, nevertheless boldly avows that he depends upon, and in turn recommends irregulars alone. To make this case still worse he says this identical individual was, but a short time since, kicked out from a respectable drug store in this city for rampant traitorous talk. Our correspondent urges that all physicians, here and elsewhere, serve him and his congeners and their wares *likewise.*

It is well to remember the scripture: Have nothing to do with the unfruitful works of the ungodly but rather reprove them. A man can not meddle with pitch without being defiled.

---

*A Theoretical and Practical Treatise on Midwifery.* Including the Diseases of Pregnancy and Parturition, and the attentions required by the child from Birth to the Period of Weaning. By P. Cazeaux, member of the Imperial Academy of Medicine; Adjunct Prof. in the Faculty of Med. of Paris, etc., etc., adopted by the Superior Council of Public Instruction, and placed by ministerial decision in the rank of the classical works designed for the use of midwife students in the Maternity Hospital of Paris. *Third American*, Translated from the Sixth French Edition, by William R. Bullock, M. D. One hundred and forty illustrations. Pp. 971, Philadelphia, Lindsay & Blakiston, 1863. [Price, $4.50.]

This magnificent work is too well known to the majority of our readers to require extended comment. As the title page denotes it is a book of classical standing and authority in France, and it must rank among the first in any language.

The author's opportunities for testing the principles of treatment, and the correctness of the doctrines put forth by other writers, have been unsurpassed, and he has brought to the touchstone of his daily experience everything which he here lays down as truth. Whilst admitting that he has collected from every source, whatever seemed to him to bear the impress of truth, he has shaped the material thus brought together into clear and simple forms.

The great popularity of the completed work both at home and abroad is a sufficient guarantee of the skill and learning exhibited in performance of the task.

In the present edition the author besides various other additions, alterations and corrections, has introduced "an article on the important subject of Tuming by external manipulation." *En passant*—we remark that we have seen at least one case that convinces us, that this point is worthy of something better than the ridicule which some parties labor to cast upon it.

The typographical execution of this edition is of the highest artistic excellence.

---

*Clinical Lectures on the Diseases of Women and Children.* By Gunning S. Bedford A. M., M. D. Prof. Obstetrics &c., Univ. of N. Y., &c., &c. *Eighth* edition, New York; Wm. Wood & Co., 1863. [Price, $2.00.]

The repeated notices we have given this work render it unnecessary to do more than simply to announce a new edition. Prof. Bedford owes it both to the profession and himself to put forth a systematic treatise on the subjects forming the title of these detached clinical lectures.

---

*The Medical Students Vade-Mecum.* A compendium of Anatomy, Physiology, Chemistry, Poisons, Materia Medica, Pharmacy, Surgery, Obstetrics, Practice of Medicine, Diseases of the Skin, &c., &c. By George Mendenhall, M. D., Prof. of Obstetrics and Diseases of Women and Children in the Medical College of Ohio, Member of the Am. Med. Ass., &c., &c., Seventh edition, revised and enlarged with two hundred and twenty-four illustrations. Philadelphia: Lindsay & Blakiston, 1863. [Price, $2.00.]

Another edition of a work which is well worthy of the success it has achieved. One of the Baconian works wherein "knowledge is digested to aphorisms;" a work not tending, as some appear to think, to superficiality but giving solidity to the whole structure of knowledge.

These things should be known, although not these only. These things should be done—others not left undone. We approve the syllabus and the epitome—none the less the elaborate treatise and cyclopædia.

---

*A Manual of Minor Surgery.* By JOHN H. PACKARD, M. D., Demonstrator of Anatomy in the University of Pennsylvania, &c., &c. With 145 Illustrations. Authorized and adopted by the Surgeon-General of the U. S. A. for the use of Surgeons in the Field and General Hospitals. Philadelphia: J. B. Lippincott & Co. 1863. pp. 288.

This work is well adapted for the use for which it was designed, and also for civil practice. Its methods and descriptions are simple, clear and methodical—well brought up to the present time.

---

*Clinical Lectures on Diseases of Women.* By J. Y. Simpson, M. D., F. R. S. E., Prof. of Midwifery in the Univ. of Edinburgh, etc., etc. Illustrated with 102 engravings on wood. *Quae prosunt ommibus.* Philadelphia: Blanchard & Lea. 1863. Pp. 510.

Thirty-eight of Prof. Simpson's Clinical Lectures are here collected and issued in permanent form. Two lectures upon Vesico Vaginal Fistula; three upon Cancer of the Uterus; one upon carcinoma of the Uterus and Mamma; two upon Dysmenorrhœa; three upon various forms of disease of the Vulva and Vagina; three upon Surgical Fever; two upon Phlegmasia Dolens; one upon coccodynia and Dis. and Deform of the coccyx; two Pelvic Cellulitis; one Pelvic Haemnatoma and Varix; two spurious Pregnancy; six upon Ovarian Dropsy; two upon Ovariotomy; one Cranioclasm, &c.; one Dropsy of Fallopian Tubes; two Puerperal Mania; one Sub-Involution of the Uterus; one Super-Involution of the Uterus and Amenorrhœa, and two lectures on Amenorrhœa conclude the series.

The profound acquirements, sound judgment and genius of the Edinburgh Professor which have given him the position of perhaps the first obstetrician of the age, are well illustrated in this work, and hence it is only needed to give the table of contents of his book to assure our readers of its great value.

*The Action of Medicines in the System.* "On the mode in which Therapentic agents introduced into the stomach produce their peculiar effects on the animal economy." Being the Prize Essay to which the Medical Society of London awarded the Fothergillian Gold Medal for MDCCCLII. By FREDERICK WILLIAM HEADLAND, M. D., B. A., F. L. S., Licentiate of the Royal College of Physicians, etc., etc. Fourth American edition. Philadelphia: Lindsay & Blakiston. 1863. Pp. 448.

This is a standard essay and should be read by every one who ventures to prescribe medicines. There is no other so clear an exposition of the subject in print.

---

*A Practical Treatise on Fractures and Dislocations.* By FRANK HASTINGS HAMILTON, A. B., A. M., M. D., Lt. Col.; Medical Inspector U. S. A.; Prof. of Military Surgery and Hygiene, and of Fractures and Dislocations in Bellevue Hospital Med. Coll.; one of the Surgeons to Bellevue Hospital, N. Y.: Prof. of Military Surgery, etc., in the Long Island College Hospital, Brooklyn; Author of a Treatise on Military Surgery. *Second Edition*, Revised and Improved. Illustrated with 285 wood-cuts. *Quae Prosunt Omnibus.* Philadelphia: Blanchard & Lea. 1863. Pp. 751.

In the Preface to this edition Prof. Hamilton remarks: "By a careful revision I have sought to render this edition, as far as possible, a faithful record of the progress of that branch of surgical science of which it treats. With this view some portions have been amended, some paragraphs have been excluded, and considerable additions have been made. The short chapter on "Gun Shot Fractures seemed to be demanded at this moment, and especially as the work has been placed upon the United States Army Supply Table for Post and General Hospitals."

This, as is universally conceded, is the most complete and valuable work on the subject extant. The first edition having been fully noticed in the Journal we have only to say that the present has been very considerably improved by the retouching and additions given it by the author. As it now stands, it is invaluable to the surgeon, whether in military or civil life.

---

*A Practical Handbook of Medical Chemistry.* By John E. Bowman, F. C. S., Formerly Prof. of Practical chemistry in King's College, London. Edited by Charles L. Bloxam, Prof. of Practical Chemistry in King's College, London. *Third* American from the *Fourth* and Revised London edition. With Illustrations. Blanchard & Lea. 1863. Pp. 351.

In the present edition all the recent advances in medical chemistry have been introduced so far as practicable, still preserving the character of the Hand Book. Among the additions may be mentioned, the quantitative determination of Ammonia and Kreatinine in the Urine, and the application of the volumetric principle to the analysis of the urine. Brief practical directions for examination of the solid excrements, of bile, liquids of muscular flesh, &c. General methods of de termining the presence of poisons in organic mixtures. The electrolytic method. Concluding with directions for the application of the elegant process of dialysis introduced by Prof. Graham, to the separation of poisons from organic mixtures-

We are gratified that the enterprising publishers have given us this new edition of this invaluable little work. Every practitioner should have one before him, before he engages in organic medical analysis, and particularly before he ventures upon the witness stand before the very inquisitive gentlemen of the bar.

---

*A Practical Treatise on Dental Medicine.* Being a compendium of medical science, as connected with the study of Dental Surgery. By Thos. E. Bond, A. M., M. D., Professor of Special Pathology and Therapeutics in the Baltimore College of Dental Surgery. Third Edition, Revised, Corrected and Enlarged. Philadelphia: Lindsay & Blakiston. 1863. Pp. 411. [$3.00.]

Time has not permitted us to give more than a cursory glance at the contents of this book, but so far as we can judge it is well adapted to the object proposed, namely, the communication to Dentists of such professional information of a general character as more immediately bears upon their specialty We can safely commend the book, already having achieved the success of a third edition, to our friends of the dental fraternity, and at the same time suggest that it contains matter deserving the attention of the general practitioner.

---

*Fibrinous Coagula in the Heart.* The fibrinous coagula occassionably found in the cavities of the heart, and which have long been considered by the greater number of authorities as merely formed *in articulo mortis*, have latterly—since the scientific investigations with reference to Thrombi and Emboli have thrown so much light on a previously obscure branch of pathology—attracted much attention from pathologists.

At a recent meeting of the London Pathological Society, Dr. Ogle exhibited eight preparations "illustrating the spontaneous formation of fibrinous coagula, at a long period before death, in the cavities of the heart, most of which had undergone considerable softening, and some of which were quite fluid in their centre. In several of these specimens the central puriform fluid was bounded by a firm smoothish surface, reminding one of the wall of an abscess, and welled out on section of the clot being made. The firm character of these coagula, their color, their adherence to the walls of the heart, and the changes which had taken place or were taking place within them, marked conclusively that the formation of the coagula had occurred sometime, possibly some weeks, before death. Out of the eight cases, this old standing and degenerating coagulum was found in the right auricle in five cases, in the right ventricle in three cases, and in the left ventricle in one case only. In almost all instances the cases had been such as included retardation of the blood's circulation through the lungs, and mostly a long and lingering death; the patients also being chiefly subjects of ill-health or intemperance."

Dr. Ogle thought pyaemia might be produced by bursting of a concretion. No special phenomena had marked the formation; a term of months might have elapsed he believed in some cases. Dr. Ogle also related some cases of embolism of both middle cerebral arteries, and of the coronary arteries.

We have frequently observed these fibrinous concretions in the cavities of the heart, and in at least one instance seen distinct vascularity of the new formation entitling it to the denomination of polypus.

Our friend Dr. J. P. Lynn exhibited to us, a few days since, a well marked specimen evidently formed long anterior to the death of a well known resident of this city.

With regard to the causes, it is evident that all that is requisite is first a nidus upon which the clot may form, then an excess of the fibrin of the blood, and lastly, but perhaps most important of all, such a degree of retardation of the velocity of the circulation as may favor deposit upon the roughened surface of the excess of fibrin present.

The physiological principles involved are well understood, and have been taken advantage of, again and again, in the cure of aneurism and stoppage of haemorrhage by position, and mechanical and medical appliances.

Propositions to which we wish now to call attention are simply and briefly these: *The increased frequency of the heart's contraction in inflammatory and febrile affections has the physiological object of preventing the formation of coagula in its cavities. Rash interference with this prophylactic effort of nature, by agents which notably retard the heart's action, directly favors formation of these coagula and their subsequent dangers.*

He who boasts that with Veratrum Viride, or Tartar Emetic, or other sedative, he has reduced the pulsation from a hundred or more, below the normal standard, by no means convinces me that, unwillingly perhaps, he is not responsible for the sudden, or it may be slow and lingering, death which ensues. In this respect, it must be confessed, Tartar Emetic is immensely superior to its proposed substitutes, because it contemporaneously with its action upon the heart, defibrinates the blood.

---

*Purulent Sinuses of the Mamma Cured by Rest.* The powerful curative agency of *rest* is well illustrated in a case of deep purulent sinuses in the left mamma of five months standing treated by Mr. Ure in St. Mary's Hospital, London. The breast was perforated in several places by the sinuses which could be traced under the gland, and from which there was a discharge of sero-purulent matter. It felt hard, somewhat fuller than the other, and was the seat of occasional throbbing pain. The difficulty was attributed to a blow on the part. Patient in a state of nervous trepidation from the idea of its cancerous nature. Mr. Ure directed the left arm to be kept steadily bandaged close to the side, with the forearm brought across the chest; the breast itself being simply covered with a piece of lint to absorb the discharge. Without other treatment the cure was complete in ten days. The principle involved is well worth application to other cases now generally treated by laying open the sinuses, or by the use of injections with uncertain result.

---

*Mercury in Syphilis.* Mercury still retains its pre-eminence in the estimation of most English surgeons in the treatment of syphilis. In a recent discussion of the subject in the Royal Med. Chirurg. Society, Dr. O'Connor laid down the proposition categorically "that what was good in the treatment of syphilis, was mercury, and what was not good was not mercury; when to use and when to discontinue it was the great desideratum in the treatment of syphilis. The insoluble preparations of mercury he believed to be the most efficient, used externally by inunction, internally, or in the form of suppositories. Iodide of Potassium he believed to be valueless as an antisyphilitic remedy, but much benefit was derived from its use after a proper mercurial treatment, in promoting the elimination from the tissues of the insoluble preparations of mercury already partially acted on by the juices of the body. Besides Iodide of Potassium was a prophylactic to tertiary symptoms."

Mr. Folly and others urged that the greater prevalence of secondary affections at the present time, was the legitimate fruit of the non-mercurial doctrines in vague.

---

*Uses of Glycerin.* E. J. Tilt, M. D. of the Farmington General Dispensary considers that Glycerin has Antiseptic properties inasmuch as it speedily gives a healthy appearance to foul, unhealthy, and even pultaceous-looking sores. It is better adapted for liniments than oil being more elegant and cleanly. Although it will not dissolve ordinary fatty matters it readily dissolves the sebaceous product of the skin, and thereby facilitates cutaneous absorption. "Its stability, cleanliness, innocuousness and antiseptic properties make it a valuable ingredient for all the variety of lotions which are applied to the inflamed or to the unhealthy mucus membranes of the mouth, eyes, nose, ears, rectum and vagina.

Starch, eighty grains, boiled in an ounce of glycerine yields a moderately stiff, tenacious plasma well adapted for all the uses of simple ointment, not becoming rancid, inodorous, not soiling the clothing and readily removable by means of a damp towel. Boiling from 100 to 150 grs. of starch to the ounce of Glycerine gives a very firm and tenacious compound well suited as a basis for medicated plasters, readily holding in solution or suspension all the ordinary ingredients of the plasters now in use.

As illustrative of the uses of the ointment Dr. Tilt gives the following formula which he frequently prescribes in the spinal and pelvic pains attendant on uterine inflammation:

℞ Sulphate of atropia, gr. ij; Glycerine, ʒ ss; Oil of Neroli, gtt. iv; Glycernie Ointment ℥j. M. A portion of this ointment about the size of a hickory nut to be well rubbed in night and morning. ℞ Acet. Morph., gr. x; Otto of Roses, gtt. j; Glycerine ʒ ss; Glycer. Oint. ℥j. M.

Instead of a Belladonna plaster he uses: ℞ Sulph. Atropia, gr. iv; Otto of Roses gtt. j; Hard Glycerine Oint., ℥j. Rub the salt, with a few drops of the Glycerine, and then incorporate all with the ointment. For a compound sedative plaster: ℞ Sulph. Atropia, gr. iij; Veratriae gr. iij; Sulph. Morphia gr. viij; Otto of Roses gtt. j; Hard Glycerine Oint ℥j. M.

---

*Removal of the Uterus and Ovaries.* Dr. Charles Clay, of Manchester, England, reports to the London Obstetrical Society "the entire extirpation of the uterus and its ovaries through the abdominal walls, which has ended most fortunately, the lady returning to her friends on the thirty-fifth day after the operation, and still continuing well, thus establishing another great fact in abdominal surgery. The case was that of a fibroid uterus of eleven pounds weight, with the ovaries in an unhealthy condition, and the tumor by its growth had latterly so filled up the cavity of the pelvis as to render the passage of the fæces and urine difficult. Dr. C. does not suppose that many uterine cases could be advisedly extirpated, but thinks some of those densely hard fibroid masses, where the constitution has not been greatly prostrated, might afford a fair prospect of cure under the knife.

---

*Fourth and Fifth Annual Report of the* Chicago Charitable Eye and Ear Infirmary, *For the year ending May* 1, 1862, *and the year ending May* 1, 1863. *Presented by the Board of Surgeons.*

The officers of this Charity number some of the leading citizens of Chicago, and it has been in successful operation nearly continuously from its origination.

TRUSTEES.—Walter L. Newberry, President; William H. Brown; Charles V. Dyer, Luther Haven, Vice Presidents; Ezra B. McCagg, Treasurer; Wm. Barry; Flavel Moseley; Samuel Stone, Secretary; Dr. John Evans; Cyrus Bentley; John H. Kinzie; Philo Carpenter.

E. L. Holmes, M. D., and E. Powell are Attending Surgeons. Prof. D. Brainard M. D., and Prof. J. W. Freer, M. D., Consulting Surgeons.

During the year ending May 1, 1862, *three hundred and ninety-seven* patients, and during the year ending May 1, 1863, *two hundred and forty-seven* patients were under treatment, making an aggregate of *one thousand two hundred and twenty-four* that have been treated since the opening of the Infirmary in 1858.

The following is a classified list of the forms of disease which have been under treatment during the past two years:

DISEASES OF THE EYE.—Wounds and Injuries 34, Conjunctivitis, simple 43, Conjunctivitis, granular 144, Conjuntivitis, neonatorum 24, Conjunctivitis, scrofulous 46, Conjunctivitis, purulent 16, Ulcer of Cornea 17, Opacity of Cornea 18, Staphlyoma of Cornea 14, Foreign Bodies on Cornea 7, Abscess of Upper Lid 6, Iritis 15, Occlusion of Pupil 9, Amaurosis 15, Cystic Tumors of Lids 6, Obstruction of Nasal Duct 13, Trichiasis 18, Inflammation of Lids 17, Strabismus 3, Cataract 12, Entropion 12, Extropion 9, Asthenopœa 13, Pterygium 6, Hydrophthalmos 3, Obliteration of Lachrymal Canals 2; Total 522.

DISEASES OF THE EAR.—Foreign Bodies in Ext. Meatus 8, Otorrhœa 26, Polypus 4, Tinnitus 3, Perforation Membrana Tympani 9, Impacted Cerumen 8, Thickening Membrana Tympani 9, Inflammation of Membrana Tympani 18, Unclassified 37; Total 122.

Of the aggregate number treated, viz., 644, *four hundred and fifty-one* were natives of foreign countries and *one hundred and ninety-three* of the United States

The Surgeons report an increasing interest in the Charity on the part of the benevolent, and congratulate the Trustees on the warm support received from the profession in the Northwest. They remark upon the importance of early attention to diseases of the eye, and the fact that the poor, through fear of the attendant expense, too often neglect procuring proper advice until blindness with its painful incidents results. A large proportionate number of the patients have been children, who without the relief thus afforded, would have been incapacitated for procurring a livelihood or even the rudiments of knowledge. The Report urges strongly and forcibly the establishment of a permanent Infirmary building, such as is to be found in several of the eastern cities. We understand that there are good grounds for believing that the friends of the Institution will soon comply with this appeal to their liberality.

The Dispensary of the Infirmary in Ewing's Block, corner of North Clark and North Water Sts. is open daily, from 11½ to 12½ o'clock, for the gratuitous treatment of the poor, afflicted with diseases of the Eye and Ear.

